# TNF-α-driven m6A modification disrupts the immunoregulatory function of MSCs by regulating HDAC5-dependent super-enhancers

**DOI:** 10.1038/s41419-025-08192-w

**Published:** 2025-12-23

**Authors:** Weihao Zhang, Jiajie Lin, Yi Zhou, Changhua Wu, Qibo Li, Junhua Chen, Yipeng Zeng, Zipeng Xiao, Huiyong Shen, Yanfeng Wu, Zepeng Su, Wenhui Yu, Zhongyu Xie

**Affiliations:** 1 Department of Orthopedics, The Eighth Affiliated Hospital of Sun Yat-Sen University, Shenzhen, China; 2 Center for Biotherapy, The Eighth Affiliated Hospital of Sun Yat-Sen University, Shenzhen, China

**Keywords:** Mesenchymal stem cells, Methylation

## Abstract

Mesenchymal stem cells (MSCs) are extensively utilised to treat inflammatory diseases because of their strong immunosuppressive functions. However, these functions are strongly affected by the inflammatory microenvironment in vivo, which limits the therapeutic effect of MSCs. The present study demonstrated that TNF-α impairs the immunosuppressive effect of MSCs on T-cell proliferation. Mechanistically, TNF-α treatment decreased the expression of the H3 deacetylase HDAC5 and then led to increased super-enhancer (SE) signals and increased expression of leukaemia inhibitory factor (LIF), which results in the dysfunction of MSCs' immunosuppressive effect. Intravenous infusion of MSCs overexpressing HDAC5 increased therapeutic efficacy in SKG mice with inflammatory arthritis. Notably, TNF-α downregulated HDAC5 by promoting WTAP-mediated m6A modification of HDAC5 mRNAs, which are subsequently regulated by YTHDF2 to reduce mRNA stability. Our results reveal a synergistic epigenetic regulatory mechanism between SEs and m6A modification of MSC immunosuppressive functions and provide a novel strategy to promote the clinical therapeutic potential of MSC infusion in inflammatory diseases.

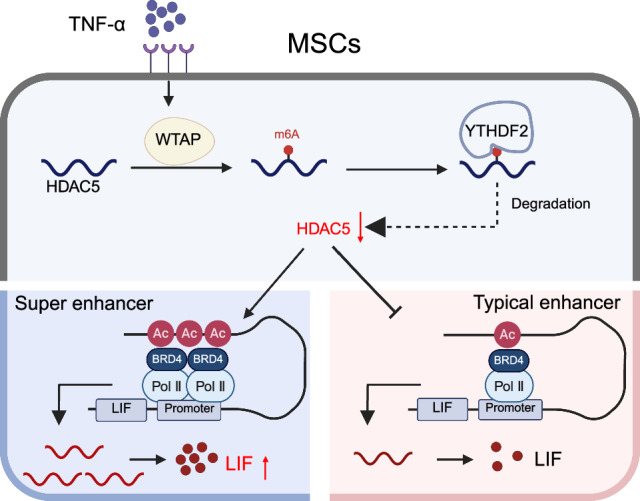

## Introduction

Mesenchymal stem cells (MSCs) are pluripotent stem cells with powerful immunoregulatory functions that have emerged as promising therapeutic agents in allogeneic cell therapy for various inflammatory diseases [1], such as graft-versus-host disease (GVHD), inflammatory bowel disease (IBD), and rheumatoid arthritis (RA). However, the functions of infused MSCs are strongly affected by the complicated inflammatory environments of these patients, thereby limiting their clinical application and resulting in a low treatment response [2, 3]. Specifically, there exists a paradox wherein an inflammatory environment is necessary to activate the immunomodulatory functions of MSCs [4], yet key mediators like TNF-α can simultaneously induce their apoptosis [5]. To provide new insight for improving MSC therapy, further investigations concerning the specific roles and mechanisms by which inflammatory factors affect the immunoregulatory functions of MSCs in vivo should be conducted.

Leukaemia inhibitory factor (LIF) is the most versatile member of the interleukin-6 cytokine family. In general, as an immunosuppressive factor, LIF can regulate various types of immune cells, including T cells, dendritic cells and macrophages [6], and is one of the critical cytokines secreted by MSCs that maintains their immunoregulatory functions. In addition, the level of secreted LIF serves as a predictor of the MSC status, indicating a positive correlation between LIF expression and the therapeutic outcome of infused MSCs in vivo [7].

Super-enhancers (SEs) consist of extensive clusters of typical enhancers (TEs) that have the ability to recruit transcription factors and cofactors at a high density to facilitate the effective transcription of target genes [8]. As a marker of SE activation, H3K27ac is regulated by histone acetylases (HATs), such as CBP/p300, and histone deacetylases (HDACs), such as HDAC5, which contribute to SE formation [9]. Previous studies have shown that SE-associated genes significantly promote the osteogenic and adipogenic differentiation of MSCs [10, 11]. However, it is still unknown whether SEs or their related molecules are involved in the immunoregulatory function of MSCs.

N6-methyladenosine (m6A) methylation is one of the most common modifications of mRNAs and is associated with the regulation of gene expression by affecting mRNA stability, precursor mRNA processing and translation [12]. Our previous study demonstrated that the ability of MSCs to recruit monocytes is regulated by m6A-mediated expression of MCP1, which modulates the in vivo immunoregulatory function and therapeutic effect of MSCs [13]. Recent studies revealed the epigenetic regulatory network between m6A and SEs in the precise regulation of various kinds of cells [14, 15]. Although epigenetic modifications are known to critically regulate MSCs' function [16], the specific impact of the m6A-SE network on their immunoregulatory capacity remains elusive.

In this study, we demonstrated that TNF-α impairs the immunosuppressive capability of MSCs through a synergistic epigenetic regulatory mechanism involving m6A modification-mediated SEs of LIF. In addition, MSCs overexpressing HDAC5 successfully prevented TNF-α-induced impairment of immunosuppressive function and improved therapeutic effects in an SKG arthritis model. Our study revealed the relationship between SEs and m6A modifications in the regulation of MSC immunoregulatory capacity and suggested a new strategy that promotes the therapeutic potential of MSCs in inflammatory diseases.

## Method

### Study approval

The collection of human peripheral blood/bone marrow and the experiments with them were approved by the Ethics Committee of the Eighth Affiliated Hospital, Sun Yat-Sen University (approval number: 2024r038). All murine experimental procedures were approved by the Institutional Animal Care and Use Committee of Sun Yat-Sen University (approval number: 2024003079). All the methods were performed in accordance with the relevant guidelines and regulations.

### Cell culture

A total of 23 donors in our study were informed of the possible risks and signed written informed consent forms, including participation in this study and the use of tissue samples. All donors who were selected for the study had no history of any significant illness. The protocol for human bone marrow MSC isolation and culture was described previously [17]. In brief, bone marrow was initially harvested from the posterior superior iliac spine, followed by the isolation and purification of MSCs using density gradient centrifugation at 12,000 rpm for 30 min. The purified MSCs were resuspended in growth medium (GM) composed of DMEM (Gibco, MA, USA) supplemented with 10% FBS (Hangzhou Sijiqing Biological Engineering Materials Co., China) and cultured at 37 °C in a 5% CO_2_ incubator. The MSCs were digested with 0.25% trypsin and subcultured into new flasks upon reaching 80–90% confluence. All MSCs used for the experiments were at passage 3.

Human CD3 + T cells were isolated from 9 healthy donors. Fifty millilitres of whole blood was collected from donors, and density gradient centrifugation was used to harvest peripheral blood mononuclear cells (PBMCs). To purify the T cells, the collected PBMCs were labelled with CD3 magnetic beads (Miltenyi Biotec, Germany) and selected via a separation column under a magnetic field. CD3+ cells were collected and immediately cultured with MSCs.

### siRNA and plasmid transfection

The specific siRNAs used were designed and synthesised by IGE Biotechnology. siRNAs targeting BRD2 (s12070), BRD3 (s15545) and BRD4 (s23901) were purchased from Ambion. For target gene knockdown in MSCs, cells were transfected with siRNAs using Lipofectamine RNAi MAX reagent (Thermo Fisher Scientific, NH, USA) following the manufacturer’s protocol. The detailed sequences of each siRNA are provided in Supplemental Table 1.

### Lentiviral construction and transfection

HDAC5-, WTAP- or YTHDF2-overexpressing lentiviruses (OE-HDAC5, OE-WTAP and OE-YTHDF2) and their vector controls (OE-NC) were designed and constructed by GeneChem Technology, and these vectors (1 × 10^8^ TU/ml) and 5 μg/ml polybrene (GeneChem, China) were used to infect MSCs at a multiplicity of infection (MOI) of 50. Further experiments were performed after 72 h.

### dCas13b-ALKBH5 lentiviral construction and transfection

The dCas13b-ALKBH5 lentiviral, which expresses a fusion protein of catalytically inactive LwaCas13b and the human m6A demethylase ALKBH5, was purchased from GeneChem Technology. For site-specific manipulation of m6A at HDAC5 mRNA, gRNA targeting the regions upstream of the predicted m6A sites on HDAC5 mRNA (gRNA-1 for site 1: TCCAAAGAAGCATGGTGGGCTCC; gRNA-2 for site 2: CAAATCCATTCTTGAGCTCTCCT) and a non-targeting control gRNA (NT-gRNA) were designed and produced by GeneChem Technology. MSCs were incubated with the lentiviruses (dCas13b or dCas13b-ALKBH5 combined with either gRNA-1, gRNA-2, or NT-gRNA) in the presence of 5 μg/ml polybrene for 24 h. Transfected MSCs were selected with 2 µg/ml puromycin. Targeted demethylation efficacy was confirmed by SELECT-qPCR.

### Coculture of MSCs and T cells

MSCs were plated at a density of 0.3 × 10^5^ cells per well in 24-well plates and exposed to TNF-α (400 ng/ml, MCE, NJ, USA), IL-17a (200 ng/ml, MCE, NJ, USA), or LPS (4 μg/ml, MCE, NJ, USA) for 48 h. Then, the supernatant was removed, and the T cells were cocultured with the MSCs at a ratio of 10:1 in RPMI 1640 (Gibco, MA, USA) containing 10% FBS, IL-2 (100 IU/ml, MCE, NJ, USA), anti-CD3 (2 μg/ml; PeproTech, NJ, USA) and anti-CD28 (2 μg/ml; PeproTech, NJ, USA) for 5 days. For gene knockdown or overexpression, MSCs were transfected with siRNA or lentiviruses before TNF-α treatment. For the cell proliferation assay, T cells were stained with carboxyfluorescein diacetate succinimidyl ester (CFSE; MCE, NJ, USA) before coculture and collected, and the CFSE fluorescence intensity was detected by flow cytometry on the 5th day.

### ELISA

A human LIF ELISA kit (RayBio, GA, USA) was used to quantify LIF in the culture supernatant. The culture supernatant was collected from each group after TNF-α or TNF-α + ZXH-3-26 treatment, added to a plate, and incubated with antibodies at 37 °C for 1 h. Culture medium supplemented with TNF-α + ZXH-3-26 served as a control. The data were processed from plate readings taken at 450 nm.

### RNA stability assay

To measure RNA stability, MSCs were seeded in a 12-well plate and processed according to experimental requirements. Then, actinomycin D (20 μg/ml; MDbio, China) was added to the medium for 0, 2, 4, or 6 h. After treatment, total RNA was immediately extracted, and RT-qPCR was performed to analyse the mRNA expression of HDAC5 at different time points.

### RNA extraction and RT-qPCR

Total RNA was harvested from MSCs via TRIzol reagent (Invitrogen, CA, USA). A total of 500 ng of RNA was subsequently reverse-transcribed into cDNA via a Prime Script TMRT reagent kit (TaKaRa, Japan). Then, qPCR was performed to analyse the expression levels of the target genes via SYBR Premix Ex Taq reagent (TaKaRa) on an Applied Biosystems 7500 Real-Time PCR System (Thermo Fisher Scientific). The relative gene expression levels were determined to be equal to the GAPDH expression level using the 2^−△△Ct^ method. The primers used to target each gene in this study are listed in Supplemental Table 2.

### Western blot

The total proteins in the MSCs were extracted via RIPA buffer lysis buffer containing 1% protease/phosphatase inhibitors and were normalised via a BCA assay kit (Thermo Fisher Scientific). SDS‒PAGE was used to separate protein extracts, which were subsequently transferred to 0.45 μm PVDF membranes (Millipore, MA, USA). After 1 h of blocking with 5% nonfat milk at room temperature, the membranes were incubated overnight at 4 °C with primary antibodies, including anti-LIF (ab138002, Abcam), anti-HDAC5 (ab55403, Abcam), anti-METTL3 (ab195352, Abcam), anti-WTAP (ab195380, Abcam), anti-METTL14 (ab309096, Abcam), anti-ALKBH5 (ab195377, Abcam), anti-FTO (ab126605, Abcam), anti-YTHDF2 (24744-1-AP, Proteintech), anti-YTHDC2 (27779-1-AP, Proteintech), anti-BRD2 (ab139690, Abcam), anti-BRD3 (ab50818, Abcam), anti-BRD4 (ab128874, Abcam), anti-IGF2BP1 (ab184305, Abcam), anti-IGF2BP2 (11601-1-AP, Proteintech), anti-IGF2BP3 (ab177477, Abcam), anti-YTHDF1 (ab252346, Abcam), anti-YTHDF3 (ab 220161, Abcam),anti-ACTIN(3700S, CST) and anti-GAPDH(5174S, CST). After 3 washes in PBST, the PVDF membranes were incubated with HRP-conjugated goat anti-mouse/rabbit secondary antibodies (1:3000, Boster, China) for 1 h at room temperature. The proteins in the immunoblots were detected via Immobilon Western Chemiluminescent HRP Substrate (Millipore). β-actin served as the reference protein. The protein bands were quantified using ImageJ software.

### SELECT detection assay

SELECT assays were performed to detect single-site m6A methylation of HDAC5 by using an Epi-SELECT m6A site identification kit (Epibiotek, China). Briefly, the relative HDAC5 mRNA levels across the experimental groups (SI-NC, SI-NC + TNF-α, and SI-WTAP + TNF-α) were quantified by RT-qPCR. Equimolar amounts of HDAC5 mRNA were subsequently subjected to reverse transcription. Single-base ligation was then performed using SELECT DNA polymerase and SELECT ligase. Finally, qPCR amplification was conducted with locus-specific primers targeting HDAC5 site 1 and site 2. The complete primer sequences are provided in Supplemental Table 2.

### RIP‒qPCR

The RIP assay was performed with a Magna RIP Kit (Millipore) according to the manufacturer’s instructions. Briefly, total RNA was extracted and fragmented into approximately 200 nt fragments. Then, the fragmented RNA was incubated with protein A/G magnetic beads conjugated with an anti-m6A antibody (68055-1-Ig, Proteintech, China), anti-YTHDF2 (24744-1-AP, Proteintech, China), or IgG overnight. The magnetic beads were subsequently collected, and the immunoprecipitated RNA was further purified for analysis via qPCR. A total of 1/10 of the total RNA fragments were used as input controls without immunoprecipitation, and the fold enrichment was conducted using the following formula: %input = 1/10 × 2^Ct [IP] ^^− Ct [input]^.

### CUT&Tag sequencing, qPCR and data analysis

CUT&Tag assay was performed using the NovoNGS CUT&Tag 3.0 High-Sensitivity Kit (NovoProtein, China) according to the manufacturer’s instructions. Approximately 1.0 × 10^5^ MSCs were collected and washed with 0.5 ml of wash buffer. ConA beads were added, and the mixture was incubated with anti-H3K27ac antibody or anti-BRD4 antibody on a rotator overnight at 4 °C. The samples were subsequently incubated with secondary antibodies for 1 h at room temperature. After washing and incubating with pAG-Tn5, MgCl_2_ was added to perform tagmentation for 1 h at 37 °C. Afterwards, the DNA was isolated via DNA extraction beads and amplified with specific primers. The PCR products were purified with DNA Clean Beads, and library quality was assessed.

CUT&Tag DNA was used to perform qPCR analysis or processed for library preparation and then sequenced on the Illumina NovaSeq platform at Novogene Science and Technology (China).

For data analysis, low-quality reads were removed by filtering raw data to remove and trim adaptors via TrimGalore (v0.6.6) with the parameters --phred33 -q 20 -stringency3. Clean reads were then mapped to the hg38 genome via Bowtie2 (version 2.5.1) with default parameters. All peaks were called by MACS2 (v2.1.1) with the parameters -p 1e-5 -g hs. DeepTools (v2.3.6.0) was used to normalise the CUT&Tag-seq data in RPKM using the bamCoverage command and to plot the heatmaps using the computeMatrix and plotHeatmap commands. IGV was used to visualise the normalised CUT&Tag-seq data.

The predicted enhancers were determined as peak regions of H3K27ac and BRD4. Constituent enhancers located within 12.5 kb were stitched together to identify SEs. Briefly, the stitched enhancers were ranked by using the ROSE algorithm with parameters of -s 12500 and −t 2000, and enhancers with signals higher than the signals with a slope of 1 on the intensity distribution plot were considered SEs. The remaining enhancers were considered TEs. Both SE- and TE-associated genes were annotated via Homer (v4.11.1) with default parameters. ClusterProfiler (v3.11) was used to perform GO (http://www.geneontology.org/) enrichment analyses of SE-associated genes.

### RNA-seq and data analysis

Total RNA from MSCs was extracted as described above. The purified mRNA was fragmented, and a cDNA library was subsequently constructed via reverse transcription and sequenced using the BGISEQ500 platform at the Beijing Genomics Institute.

For data analysis, raw data were filtered with SOAPnuke (v1.5.2) by removing adaptors and low-quality reads. Clean reads were mapped to the hg38 genome via HISAT2 (v2.0.4) and then aligned to the hg38 reference via Bowtie (v2.2.5). Differential expression gene (DEG) analysis was performed via DESeq2 (version 1.4.5) with |log2FC| > 1 and a *Q* value < 0.05. Heatmap, volcano plot, KEGG (https://www.kegg.jp/), and GO and GSEAs enrichment analyses of DEGs were performed via Dr Tom (BGI).

### Animal experiment

SKG mice were purchased from CLEA Japan. For SKG arthritis model induction, ten-week-old female SKG mice were administered a single intraperitoneal injection of 3 mg of curdlan. The mice were randomly divided into 3 groups: a group treated with PBS, a group treated with OE-NC MSCs and a group treated with OE-HDAC5 MSCs (each group contained 10 mice). OE-NC or OE-HADC5 MSCs (single dose, 10^7^/kg) were injected intravenously into the OE-NC or OE-HDAC5 group, respectively, on the day after induction, whereas the PBS group was treated with equal amounts of PBS. The clinical arthritis scores were measured weekly. The definite score criteria were as described previously [18]: 0 = no swelling or redness, 0.1 = swelling or redness of a digit, 0.5 = mild swelling and/or redness of the wrist or ankle joints, and 1 = severe swelling of the larger joints. Eight weeks after induction, the mice were euthanised, and the hindlimbs were collected for further experiments.

### Micro-CT scanning

The hindlimbs from SKG mice were fixed in 4% paraformaldehyde for 24 h. High-resolution micro-CT scanning with a resolution of 19 μm was subsequently performed using a Siemens Inveon CT scanner.

### Histological staining

After fixation in 4% paraformaldehyde, the hind paws were decalcified in rapid decalcification solution and embedded in paraffin. Ankle sections were prepared and stained with haematoxylin and eosin (HE) to assess inflammatory infiltration in individuals with arthritis and with safranin O to assess cartilage damage.

### Statistics

All data are representative of at least 9 independent experiments and are presented as the mean ± SD of biological replicates. GraphPad Prism 8.0 software was used to perform the statistical analysis. Two-group comparisons were performed via unpaired two-tailed Student’s *t*-tests, and three or more different group comparisons were performed via one-way analysis of variance (ANOVA) followed by Tukey’s multiple comparison tests. The investigators were not blinded to the group allocation when the outcomes were assessed. The data are expressed as the mean ± SD. We regarded *P* values < 0.05 as statistically significant, ^*^P < 0.05, ^**^P < 0.01, and ^***^P < 0.001.

## Results

### TNF-α suppresses the immunoregulatory effects of MSCs on T cells via SEs

**Fig. 1:**
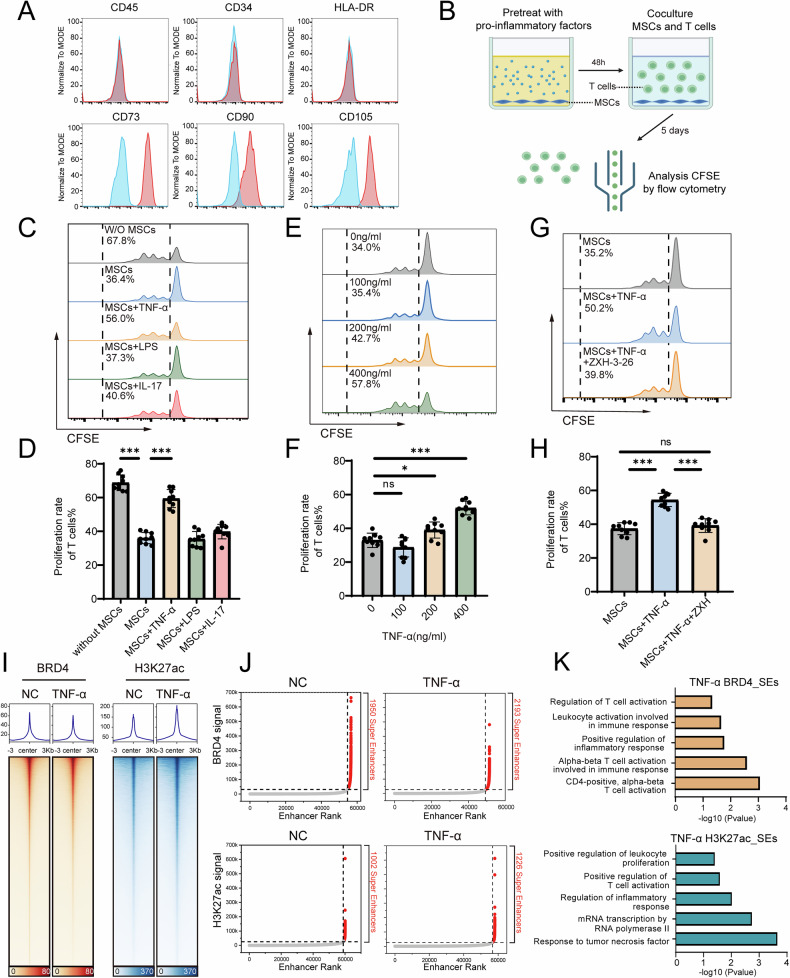
SEs mediate the TNF-α-mediated inhibition of the immunosuppressive capacity of MSCs. **A** Verification of bone marrow MSC surface markers. **B** Schematics demonstrating the workflow of the MSC-T-cell coculture system. **C** Proliferation rates of T cells alone or cocultured with MSCs treated with TNF-α, IL-17a, or LPS. **D** Quantification of the T-cell proliferation rates in (**C**). **E** Proliferation rates of T cells cocultured with MSCs treated with TNF-α at concentrations of 0, 100, 200, and 400 ng/ml. **F** Quantification of the T-cell proliferation rates in (**E**). **G** Proliferation rates of T cells cocultured with MSCs treated with TNF-α or TNF-α + ZXH-3-26. **H** Quantification of the T-cell proliferation rates in (**G**). **I** CUT&Tag heatmaps showing the BRD4 and H3K27ac signals of MSCs treated with or without TNF-α. **J** Identification of BRD4 and H3K27ac SEs in MSCs treated with or without TNF-α. **K** GO enrichment analyses of SEs in MSCs treated with TNF-α. The data are presented as the means ± SDs, *n* = 9, Tukey’s multiple comparison test, ^*^P < 0.05, ^**^P < 0.01, ^***^P < 0.001.

Human bone marrow MSCs were separated from bone marrow and verified by flow cytometry, which revealed positive results for CD73, CD90, and CD105 and negative results for CD45, CD34, and HLA-DR (Fig. 1A). We treated MSCs with several common inflammatory cytokines, including TNF-α, IL-17a and lipopolysaccharide (LPS), followed by coculture with activated T cells to investigate the impact of the inflammatory environment on the immunoregulation of MSCs (Fig. 1B). The results revealed that MSCs significantly suppressed the proliferation of activated T cells, and compared with that of untreated MSCs, the proliferation of T cells was significantly greater when they were cocultured with TNF-α-pretreated MSCs, indicating that TNF-α impaired the immunosuppressive capability of MSCs (Fig. 1C, D). This effect increased with increasing TNF-α concentration (Fig. 1E, F). A previous study showed that TNF-α could induce MSC apoptosis [19], which may explain why TNF-α impaired the immunosuppressive capability of MSCs. However, we found that TNF-α did not significantly induce the apoptosis of MSCs (Fig. S1A). Given the potential role of SEs in the regulation of MSC functions, we postulated that SEs may be involved in TNF-α-mediated immunoregulatory dysfunction in MSCs. We used ZXH-3-26, an inhibitor that selectively induces BRD4 degradation rather than BRD2/3 degradation, to suppress the dynamic distribution of SEs [20]. ZXH-3-26-treated MSCs effectively reversed the decrease in the immunosuppressive function of MSCs induced by TNF-α treatment (Fig. 1G, H). In addition, we also found that the knockdown of BRD4 but not BRD2/3 in MSCs reversed this effect (Fig. S2C, D), indicating the important role of BRD4-mediated SEs. To further explore the dynamics of SEs during TNF-α treatment, we employed a cleavage under targets and tagmentation (CUT&Tag) assay to assess 2 SE markers, H3K27ac and BRD4, in MSCs with (TNF-α group) or without (normal control, NC group) TNF-α treatment. The heatmap shows the average signals of H3K27ac and BRD4 following TNF-α stimulation, indicating distinct landscapes of enhancers in MSCs treated with TNF-α (Fig. 1I). The ROSE algorithm was subsequently used to rank all the enhancers and identified 962 SEs in the NC group and 1181 SEs in the TNF-α group on the basis of H3K27ac signals and 1950 SEs in the NC group and 2103 SEs in the TNF-α group on the basis of BRD4 signals (Fig. 1J). Gene Ontology (GO) functional analysis revealed that SEs in the TNF-α group were enriched in terms including positive regulation of the inflammatory response, positive regulation of leucocyte proliferation and regulation of T-cell activation (Fig. 1K). These findings strongly suggest that SEs are crucial in the impaired immunosuppressive function of MSCs induced by TNF-α.

### SE-driven LIF expression is involved in the impaired immunosuppressive capacity of MSCs mediated by TNF-α

**Fig. 2:**
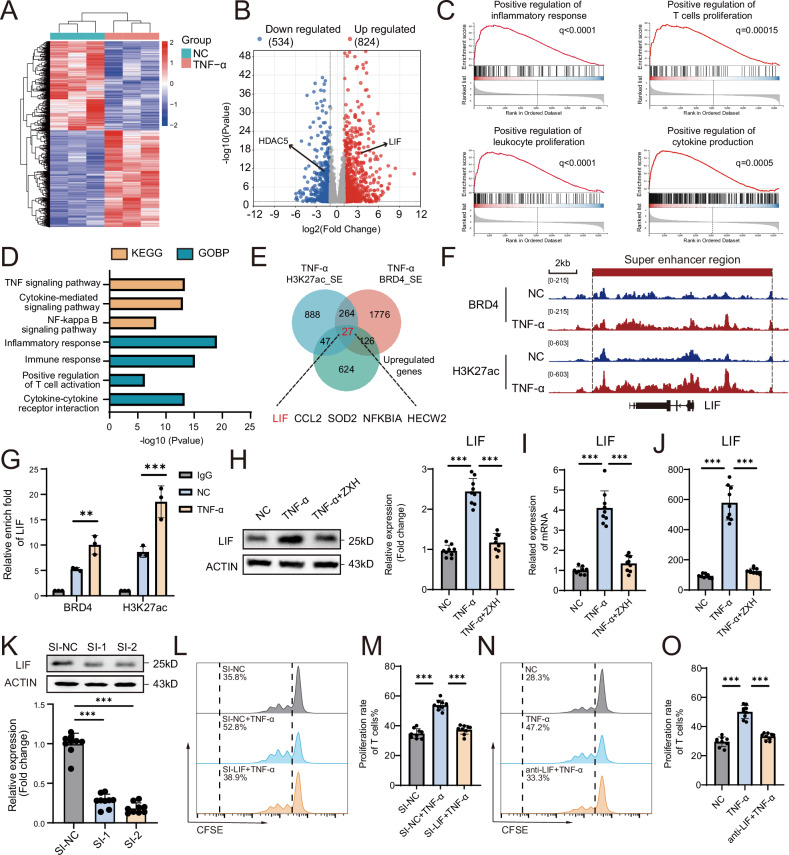
LIF upregulation in MSCs treated with TNF-α disrupts their immunoregulatory capacity. **A** The RNA-seq heatmap shows the distinct expression patterns of the NC (MSCs treated without TNF-α) and TNF-α (MSCs treated with TNF-α) groups. **B** RNA-seq volcano plot of the genes differentially expressed between the NC and TNF-α groups. **C** GSEA of the RNA-seq data between the NC and TNF-α groups. **D** KEGG and GO enrichment analyses of the genes differentially expressed between the NC and TNF-α groups. **E** The Venn diagram illustrates the intersection of H3K27ac SEs and BRD4 SEs, along with the upregulated genes in the TNF-α group. **F** Signal traces of the LIF H3K27ac and BRD4 SE regions in the NC and TNF-α groups. **G** CUT&Tag-qPCR revealed increased signals of BRD4 and H3K27ac at the LIF locus in the TNF-α group. **H** Western blot analysis showing the LIF expression levels in MSCs treated with no TNF-α, TNF-α alone, or TNF-α + ZXH-3-26. **I** qPCR analysis showing the LIF mRNA expression levels in MSCs treated with no TNF-α, TNF-α alone, or TNF-α + ZXH-3-26. **J** ELISA showing the levels of secreted LIF in MSCs treated with no TNF-α, TNF-α alone, or TNF-α + ZXH-3-26. **K** Western blot analysis showing the LIF expression levels in MSCs treated with SI-NC, SI-1 and SI-2 for LIF to verify the knockdown efficacy of the SI-RNAs. **L** Proliferation rates of T cells cocultured with MSCs treated with SI-NC, SI-NC + TNF-α or SI-LIF + TNF-α. **M** Quantification of T-cell proliferation rates in (**L**). **N** Proliferative rates of T cells cocultured with MSCs treated with no TNF-α, TNF-α alone, or TNF-α+anti-LIF antibody. **O** Quantification of T-cell proliferation rates in (**N**). The data are presented as the means ± SDs; two-tailed Student’s t-tests and Tukey’s multiple comparison tests, *n* = 9; ^*^P < 0.05, ^**^P < 0.01, ^***^P < 0.001.

To verify the key targets of SEs in this process, we performed RNA-seq on MSCs with or without TNF-α treatment. The heatmap revealed distinct gene expression profiles between the NC group and the TNF-α group (Fig. 2A). After TNF-α treatment, a total of 1358 DEGs were identified, with 824 upregulated genes and 534 downregulated genes (Fig. 2B). Gene set enrichment analysis (GSEA) of the RNA-seq data revealed significant enrichment of genes related to positive regulation of inflammation, T-cell proliferation, leucocyte proliferation and cytokine production (Fig. 2C). Furthermore, several terms, including the TNF signalling pathway, cytokine-mediated signalling pathway, inflammatory response, immune response and positive regulation of T-cell activation, were enriched in the GO and Kyoto Encyclopaedia of Genes and Genomes (KEGG) analyses (Fig. 2D). Only 27 common genes were identified by intersecting TNF-gain SEs in both H3K27ac and BRD4 and the upregulated genes of the TNF-α group from the RNA-seq data (Fig. 2E). Among the 27 candidate genes, LIF has been implicated in the modulation of T-cell proliferation and differentiation [6]. The CUT&Tag signal plot revealed an SE region of the LIF gene locus after TNF-α treatment (Fig. 2F). CUT&Tag-qPCR further confirmed these changes, revealing the presence of SEs on LIF and the increase in SE signals after TNF-α treatment (Fig. 2G). We verified the augmentation of LIF expression in MSCs exposed to TNF-α at both the mRNA and protein levels and further revealed that ZXH-3-26 reversed the upregulation of LIF induced by TNF-α (Fig. 2H, I). Given that LIF acts as a secreted cytokine, we also measured the level of LIF in the supernatant via enzyme-linked immunosorbent assay (ELISA), which revealed that the amount of LIF secreted by MSCs increased with TNF-α treatment and decreased after ZXH-3-26 treatment (Fig. 2J). To verify the role of LIF in the immunosuppression of T-cell proliferation in MSCs, we designed specific LIF siRNAs and confirmed their knockdown efficacy (Fig. 2K). Knockdown of LIF in TNF-α-treated MSCs substantially increased their ability to inhibit T-cell proliferation (Fig. 2L, M). In addition, we added an anti-LIF antibody to neutralise LIF in the MSC-T-cell coculture system and found that the ability of TNF-α to impair the immunosuppressive capacity of MSCs was rescued by the anti-LIF antibody (Fig. 2N, O). To determine whether LIF exerts its function directly on T cells or indirectly through MSCs, recombinant LIF protein was added directly to the T cell culture medium. The results showed that LIF can directly promote T cell proliferation. (Fig. S3A). In addition, we detected the immunosuppressive capacity of MSCs pretreated with LIF and found no significant difference in the suppression of T-cell proliferation or the expression of TGF-β, IL-10, ICAM-1, IDO and PD-L1 after LIF pretreatment (Fig. S3B, C). These results indicate that the negative impact of TNF-α on MSC immunoregulatory capacity is mediated by the SE-driven upregulation of LIF.

### TNF-α induces the redistribution of SEs by inhibiting the HDAC5-mediated deacetylation of H3K27ac

**Fig. 3:**
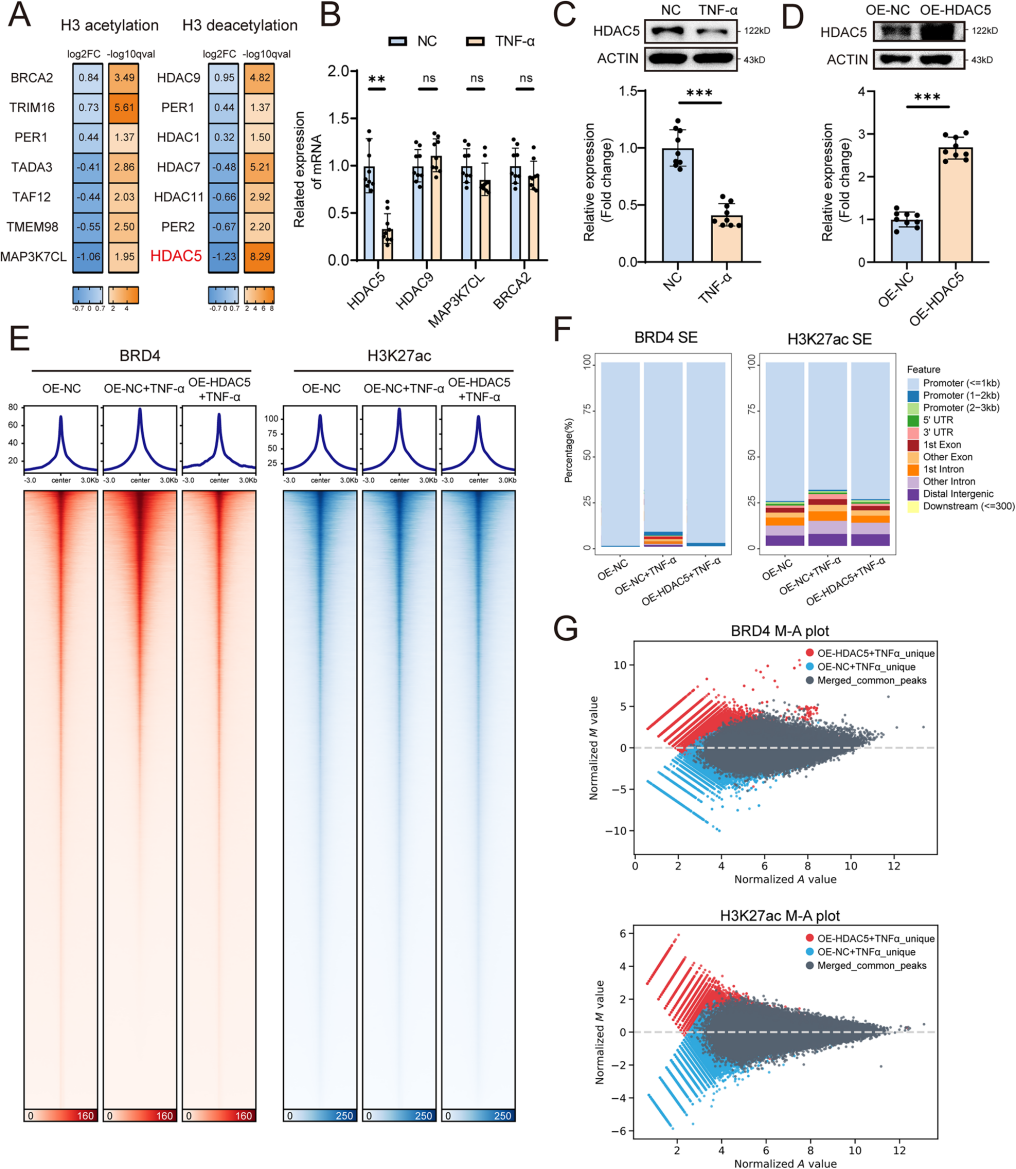
TNF-α-induced upregulation of HDAC5 regulates the dynamic distribution of SEs in MSCs. **A** Expression of genes associated with H3 acetylation and deacetylation. **B** qPCR analysis showing the mRNA expression levels of HDAC5, HDAC9, MAP3K7CL and BRCA2. **C** Western blot analysis showing the HDAC5 expression levels in MSCs treated with or without TNF-α. **D** Western blot analysis showing the HDAC5 expression levels in MSCs treated with OE-NC or OE-HDAC5. **E** CUT&Tag heatmaps showing the BRD4 and H3K27ac signals of MSCs treated with OE-NC, OE-NC + TNF-α or OE-HDAC5 + TNF-α. **F** BRD4 and H3K27ac SE distributions among the genomic elements of MSCs treated with OE-NC, OE-NC + TNF-α or OE-HDAC5 + TNF-α. **G** Differentially enriched peaks between MSCs treated with OE-NC + TNF-α or OE-HDAC5 + TNF-α. The data are presented as the means ± SDs; two-tailed Student’s *t*-tests, *n* = 9; ^*^P < 0.05, ^**^P < 0.01, ^***^P < 0.001.

**Fig. 4:**
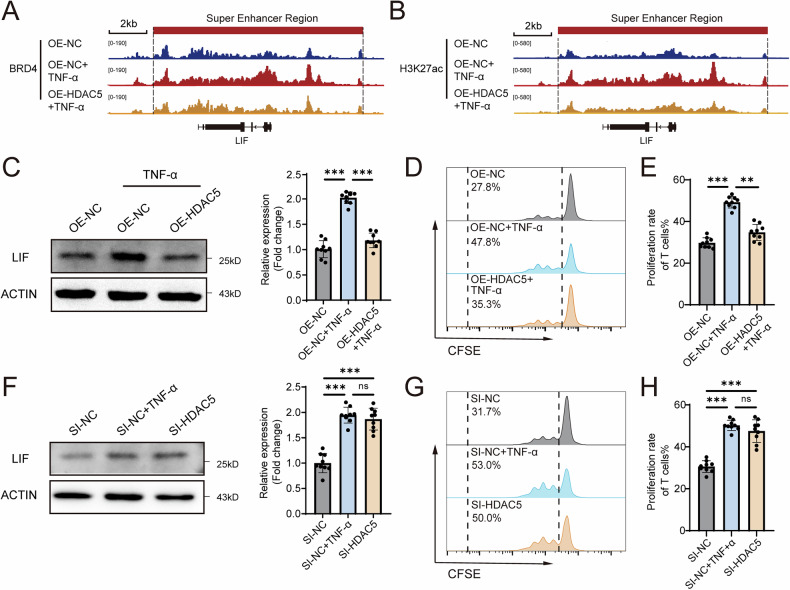
TNF-α increases LIF expression by impairing HDAC5-mediated SE redistribution. **A** Signal traces of LIF BRD4 SE regions in MSCs treated with OE-NC, OE-NC + TNF-α or OE-HDAC5 + TNF-α. **B** Signal traces of LIF H3K27ac SE regions in MSCs treated with OE-NC, OE-NC + TNF-α or OE-HDAC5 + TNF-α. **C** Western blot analysis showing the LIF expression levels in MSCs treated with OE-NC, OE-NC + TNF-α or OE-HDAC5 + TNF-α. **D** Proliferation rates of T cells cocultured with MSCs treated with OE-NC, OE-NC + TNF-α or OE-HDAC5 + TNF-α. **E** Quantification of the T-cell proliferation rates in (**D**). **F** Western blot analysis showing the LIF expression levels in MSCs treated with SI-NC, SI-NC + TNF-α or SI-HDAC5. **G** Proliferation rates of T cells cocultured with MSCs treated with the SI-NC, SI-NC + TNF-α or SI-HDAC5. **H** Quantification of the T-cell proliferation rates in (**G**). The data are presented as the means ± SDs; Tukey’s multiple comparison tests, *n* = 9; ^*^P < 0.05, ^**^P < 0.01, ^***^P < 0.001.

As an important marker of enhancers, the dynamic level of H3K27ac has been reported to affect the distribution of SEs [21]. Given the significant changes in SE signals after TNF-α treatment, we investigated the regulators of H3K27ac involved in TNF-α-mediated SE distribution. RNA-seq analysis revealed that the expression of the H3 deacetylase HDAC5 was most notably altered among the genes related to H3K27ac modification (Fig. 3A). We subsequently verified the significant downregulation of HDAC5 expression at both the mRNA and protein levels after TNF-α treatment (Fig. 3B, C). To determine whether HDAC5 affects SEs in MSCs, we established MSCs overexpressing HDAC5 via lentiviral transduction (Fig. 3D) and then performed a CUT&Tag assay to analyse the distribution and intensity changes in H3K27ac and BRD4 signals following HDAC5 overexpression. We found that TNF-α treatment significantly altered the distribution of SEs in MSCs, while the overexpression of HDAC5 reversed these changes (Fig. 3E, F). Additionally, the overexpression of HDAC5 in TNF-α-treated MSCs significantly altered the BRD4 and H3K27ac peaks (Fig. 3G). Moreover, the overexpression of HDAC5 in MSCs strongly reversed the increase in SE signals and upregulated the expression of LIF (Fig. 4A–C). The impaired immunosuppressive ability of MSCs treated with TNF-α was also reversed after HDAC5 overexpression (Fig. 4D, E). Interestingly, MSCs with HDAC5 knockdown but without TNF-α treatment presented a phenotype similar to that of TNF-α-treated MSCs, including the upregulation of LIF expression and impaired suppression of T-cell proliferation (Fig. 4F–H). Taken together, these results suggest that TNF-α-mediated inhibition of HDAC5 expression leads to increased SE signalling and expression of LIF, thereby contributing to the impaired immunosuppressive capacity of MSCs.

### Infusion of MSCs overexpressing HDAC5 promotes therapeutic effects on inflammatory arthritis

**Fig. 5:**
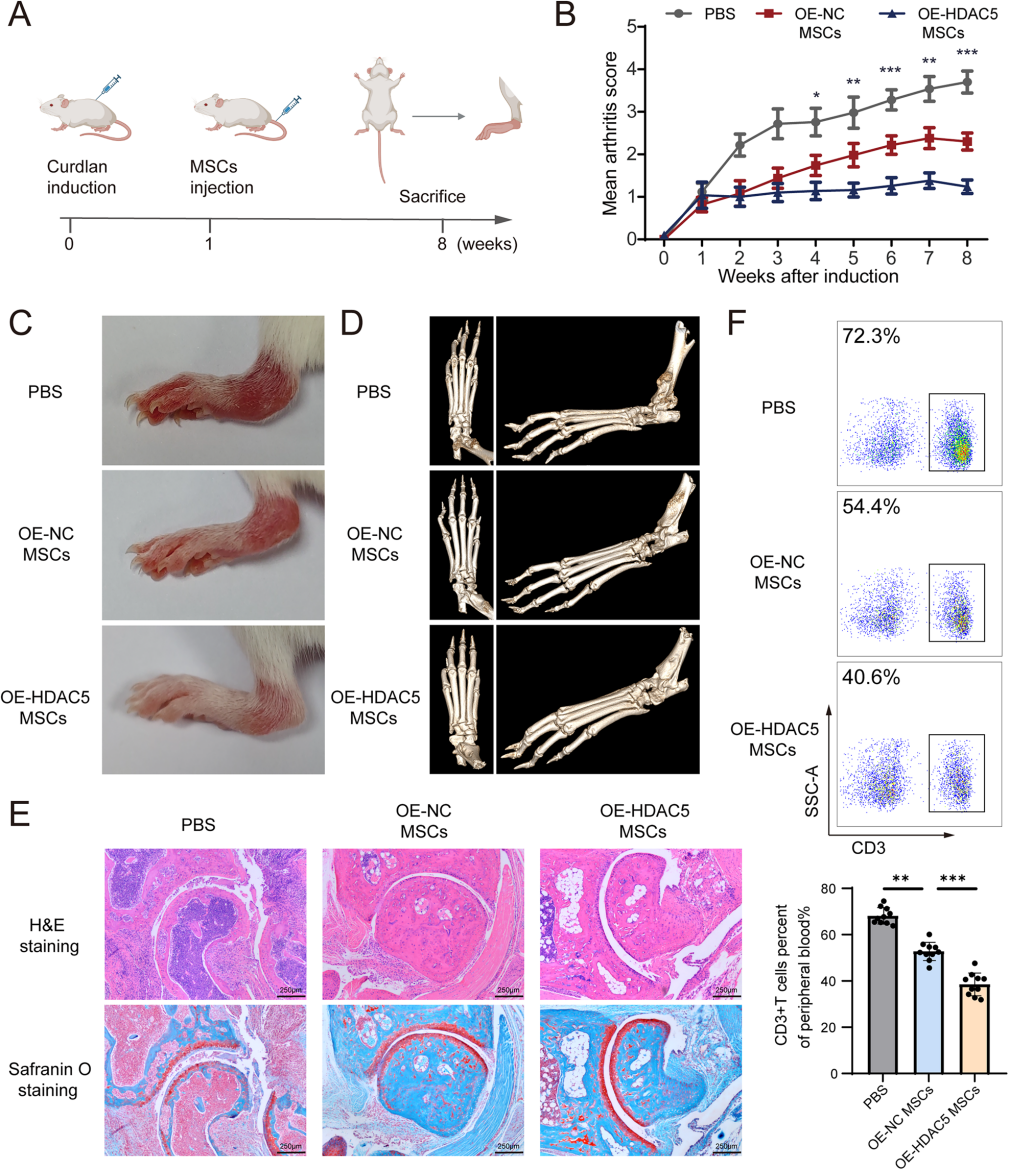
Infusion of MSCs overexpressing HDAC5 optimises the therapeutic effects on inflammatory arthritis. **A** Schematic showing the workflow of arthritis induction, MSC infusion and analysis of the therapeutic effect. **B** Arthritis scores of SKG mice intravenously injected with PBS, OE-NC MSCs or OE-HDAC5 MSCs. **C** Images showing the ankle swelling of SKG mice intravenously injected with PBS, OE-NC-MSCs or OE-HDAC5-MSCs. **D** Micro-CT images of the osteophytes in SKG mice intravenously injected with PBS, OE-NC MSCs or OE-HDAC5 MSCs. **E** H&E staining and safranin O staining of the ankles of SKG mice intravenously injected with PBS, OE-NC-MSCs or OE-HDAC5-MSCs. **F** CD3 + T-cell proportions in the peripheral blood of SKG mice intravenously injected with PBS, OE-NC-MSCs or OE-HDAC5-MSCs. The data are presented as the means ± SDs; Tukey’s multiple comparison tests, *n* = 9; ^*^P < 0.05, ^**^P < 0.01, ^***^P < 0.001.

After elucidating the mechanism through which TNF-α impairs the immunosuppressive function of MSCs in vitro, we developed OE-HDAC5 MSCs for treating inflammatory diseases in vivo. SKG mice, which exhibit arthritis along with high levels of TNF-α after intraperitoneal injection of curdlan [22], were generated and then intravenously injected with OE-NC MSCs or OE-HDAC5 MSCs (Fig. 5A). Compared with those in the PBS group, the severity of joint swelling and arthritis score was significantly lower in the OE-NC MSC group, whereas the therapeutic efficacy in the OE-HDAC5-MSC group surpassed that in the OE-NC-MSC group (Fig. 5B, C). Micro-CT revealed that OE-HDAC5 MSC treatment effectively protected against bone erosion and reduced the formation of ectopic bone (Fig. 5D). Both the OE-NC MSC and OE-HDAC5 MSC groups showed significant improvements in HE staining and safranin-O staining, with the latter showing more obvious amelioration of inflammatory infiltration and cartilage damage (Fig. 5E). Additionally, we tested the proportion of CD3 + T cells in the peripheral blood and found that, compared with the OE-NC MSC group, the OE-HDAC5 MSC group presented a more pronounced reduction in CD3 + T cells (Fig. 5F). Overall, these results indicate that HDAC5-overexpressing MSCs have strong therapeutic potential for arthritis in an inflammatory microenvironment.

### WTAP-m6A modification accelerates HDAC5 mRNA degradation

**Fig. 6:**
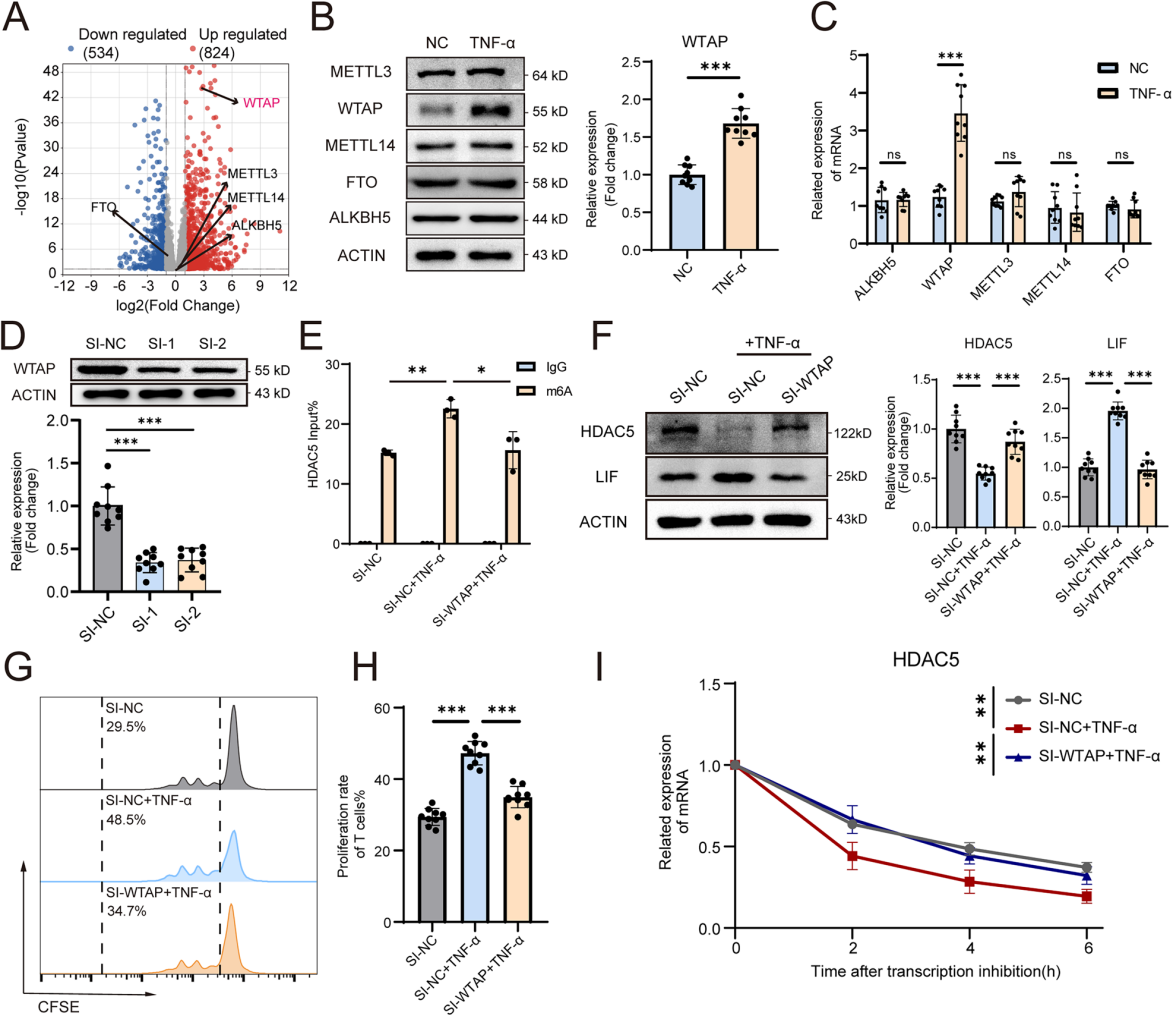
WTAP facilitates the m6A modification of HDAC5 mRNA, leading to a decrease in its expression. **A** RNA-seq volcano plot of the genes differentially expressed between the NC and TNF-α groups, showing the expression of m6A-associated genes. **B** Western blot analysis showing METTL3, WTAP, METTL14, FTO and ALKBH5 expression levels in MSCs treated with or without TNF-α. **C** qPCR analysis showing the mRNA expression levels of METTL3, WTAP, METTL14, FTO and ALKBH5 in MSCs treated with or without TNF-α. **D** Western blot analysis showing the WTAP expression levels in MSCs treated with SI-NC, SI-1 or SI-2. **E** RIP‒qPCR analysis of HDAC5 m6A methylation levels in MSCs treated with SI-NC, SI-NC + TNF-α or SI-WTAP + TNF-α. **F** Western blot analysis showing the HDAC5 and LIF expression levels in MSCs treated with SI-NC, SI-NC + TNF-α or SI-WTAP + TNF-α. **G** Proliferation rates of T cells cocultured with MSCs treated with SI-NC, SI-NC + TNF-α or SI-WTAP + TNF-α. **H** Quantification of the T-cell proliferation rates in (**G**). **I** Degradation rates of HDAC5 mRNA in MSCs treated with SI-NC, SI-NC + TNF-α or SI-WTAP + TNF-α. The data are presented as the means ± SDs; two-tailed Student’s *t*-tests and Tukey’s multiple comparison tests, *n* = 9; ^*^P < 0.05, ^**^P < 0.01, ^***^P < 0.001.

We next investigated how TNF-α regulates the expression of HDAC5. Hyperinflammation-mediated m6A modification reportedly leads to cell dysfunction [23]. A volcano plot of the RNA-seq data revealed the expression profiles of enzymes related to m6A methylation or demethylation in MSCs after TNF-α stimulation, indicating that WTAP, rather than other m6A mediators such as FTO, METTL3, ALKBH5 and METTL14, was significantly upregulated in the TNF-α group (Fig. 6A). qPCR and Western blot results confirmed the significantly increased expression of WTAP at both the mRNA and protein levels, whereas METTL3, METTL14, ALKBH5, and FTO expression was not significantly altered in TNF-α-treated MSCs (Fig. 6B, C). To explore the role of WTAP in regulating HDAC5 expression, we knocked down WTAP in MSCs via RNA interference (Fig. 6D), and m6A RNA immunoprecipitation–qPCR (RIP‒qPCR) was employed to assess the m6A modification level of HDAC5 mRNA. We found that TNF-α significantly increased the m6A modification level on HDAC5 mRNA, whereas WTAP inhibition effectively reversed this effect, which indicated that WTAP was the key m6A methyltransferase responsible for the m6A modification of HDAC5 (Fig. 6E). In addition, knocking down WTAP strongly reversed the TNF-α-induced decrease in HDAC5 expression and increase in LIF expression (Fig. 6F). WTAP knockdown also promoted the inhibitory effect on T-cell proliferation in TNF-α-treated MSCs (Fig. 6G, H). After treatment with actinomycin D to inhibit RNA transcription, the stability of HDAC5 mRNA in TNF-α-treated MSCs was significantly reduced, whereas compared with that in the TNF-α group, RNA degradation in the WTAP-knockdown group was lower (Fig. 6I). Intriguingly, we found that overexpression of WTAP in MSCs alone led to a similar phenotype to that of MSCs treated with TNF-α, including increased LIF-SE signals of H3K27ac and BRD4 (Fig. S4B), downregulation of HDAC5, upregulation of LIF (Fig. S4C) and impaired immunosuppressive capability (Fig. S4D). In addition, a greater degradation rate of HDAC5 mRNA was observed in OE-WTAP MSCs (Fig. S4E). Collectively, these results suggest that TNF-α increases WTAP, promotes the m6A modification of HDAC5 and facilitates its degradation, which further impacts SE formation and immunosuppressive ability.

### YTHDF2 binds specific m6A sites to promote HDAC5 mRNA degradation induced by TNF-α

**Fig. 7:**
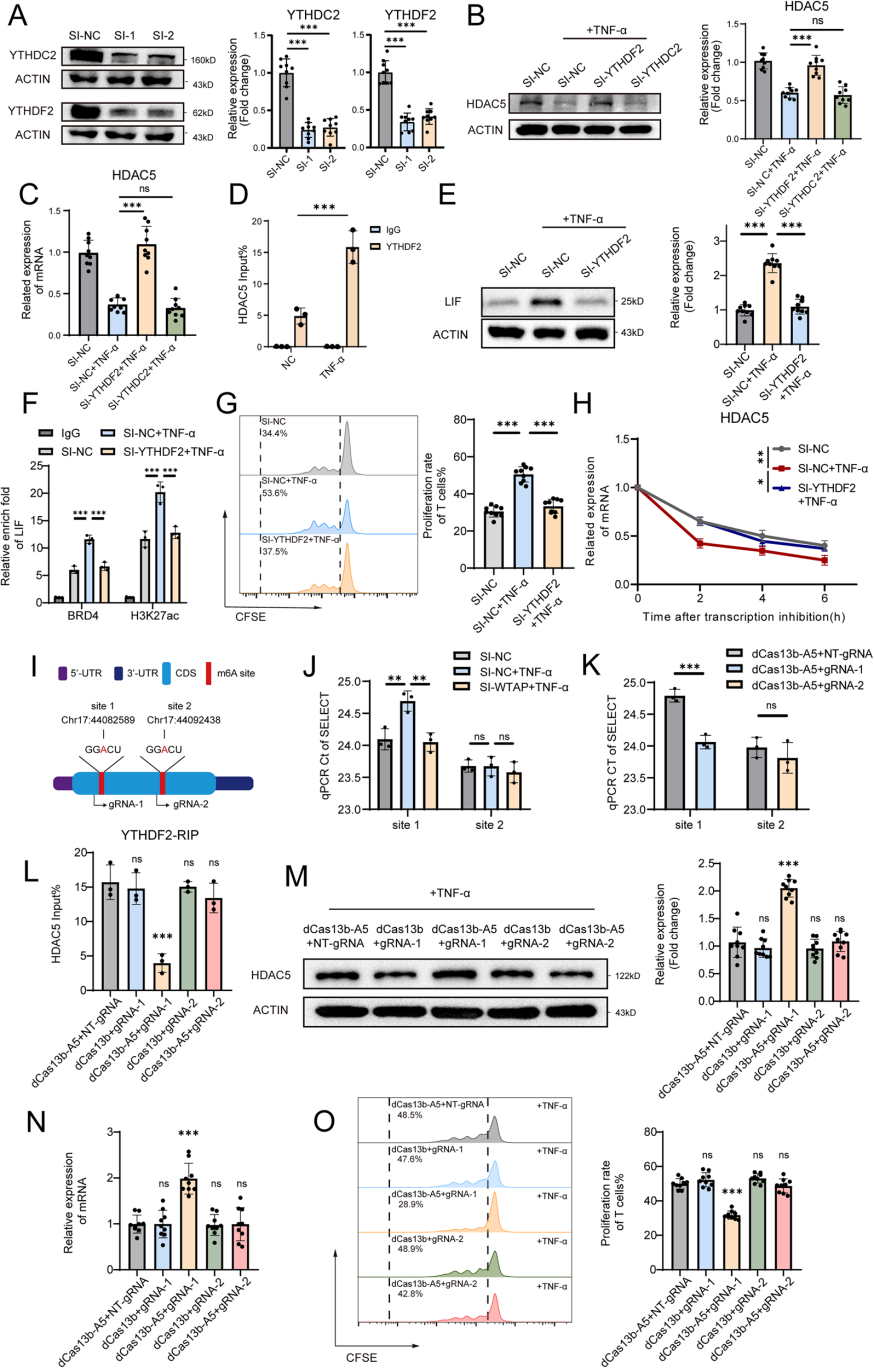
YTHDF2 binds m6A methylation sites on HDAC5 mRNA to facilitate its degradation. **A** Western blot analysis showing the YTHDC2 and YTHDF2 expression levels in MSCs treated with SI-NC, SI-1 or SI-2 for YTHDC2 or YTHDF2. **B** Western blot analysis showing the HDAC5 expression levels in MSCs treated with SI-NC, SI-NC + TNF-α, SI-YTHDF2 + TNF-α or SI-YTHDC2 + TNF-α. **C** qPCR analysis showing the mRNA expression levels of HDAC5 in MSCs treated with SI-NC, SI-NC + TNF-α, SI-YTHDF2 + TNF-α or SI-YTHDC2 + TNF-α. **D** RIP-qPCR analysis showing the binding levels of YTHDF2 to HDAC5 mRNA in MSCs treated with or without TNF-α. **E** Western blot analysis showing the LIF expression levels in MSCs treated with SI-NC, SI-NC + TNF-α or SI-YTHDF2 + TNF-α. **F** CUT&Tag-qPCR showing signals of BRD4 and H3K27ac at the LIF locus in MSCs treated with SI-NC, SI-NC + TNF-α or SI-YTHDF2 + TNF-α. **G** Proliferation rates of T cells cocultured with MSCs treated with SI-NC, SI-NC + TNF-α or SI-YTHDF2 + TNF-α. **H** Degradation rates of HDAC5 mRNA in MSCs treated with SI-NC, SI-NC + TNF-α, or SI-YTHDF2 + TNF-α. **I** Schematic representation of m6A sites of HDAC5 mRNA and corresponding gRNA targeting sites. **J** SELECT-qPCR analysis of HDAC5 m6A methylation levels at sites 1 and 2 in MSCs treated with SI-NC, SI-NC + TNF-α or SI-WTAP + TNF-α. **K** SELECT-qPCR analysis of HDAC5 m6A methylation levels at sites 1 and 2 in TNF-α-treated MSCs transfected with dCas13b-A5 + NT-gRNA, dCas13b-A5 + gRNA-1 or dCas13b-A5 + gRNA-2. **L** RIP‒qPCR analysis showing the binding levels of YTHDF2 to HDAC5 mRNA in TNF-α-treated MSCs transfected with dCas13b-A5 + NT-gRNA, dCas13b+gRNA-1, dCas13b-A5+gRNA-1, dCas13b+gRNA-2 or dCas13b-A5+ gRNA-2. **M** Western blot analysis showing the HDAC5 expression levels in TNF-α-treated MSCs transfected with dCas13b-A5 + NT-gRNA, dCas13b + gRNA-1, dCas13b-A5 + gRNA-1, dCas13b + gRNA-2 or dCas13b-A5 + gRNA-2. **N** qPCR analysis showing the mRNA expression levels of HDAC5 in TNF-α-treated MSCs transfected with dCas13b-A5 + NT-gRNA, dCas13b + gRNA-1, dCas13b-A5 + gRNA-1, dCas13b + gRNA-2 or dCas13b-A5 + gRNA-2. **O** Proliferation rates of T cells cocultured with TNF-α-treated MSCs transfected with dCas13b-A5 + NT-gRNA, dCas13b + gRNA-1, dCas13b-A5 + gRNA-1, dCas13b+gRNA-2 or dCas13b-A5 + gRNA-2. The data are presented as the means ± SDs; two-tailed Student’s *t*-tests and Tukey’s multiple comparison tests, *n* = 9; ^*^P < 0.05, ^**^P < 0.01, ^***^P < 0.001.

m6A reader proteins dictate the fate of m6A-modified mRNA transcripts. YTHDC2 and YTHDF2 are both key m6A readers responsible for m6A-mediated mRNA destabilisation [24, 25]. To investigate which of these m6A readers are responsible for the stability of HDAC5 mRNA, we knocked down YTHDF2 and YTHDC2 via siRNA transfection and confirmed the knockdown efficacy (Fig. 7A). After the expression of YTHDF2 was knocked down, the expression of HDAC5 in the MSCs treated with TNF-α was restored, while the expression of YTHDC2 was not significantly changed (Fig. 7B, C). Additionally, knockdown of other m6A readers, such as IGF2BP1/2/3, YTHDF1/3 and YTHDC1, was unable to restore HDAC5 expression (Fig. S5A, B), indicating that YTHDF2 is likely the critical m6A reader in this process. The RIP-qPCR results suggested that HDAC5 mRNA was effectively immunoprecipitated using an anti-YTHDF2 antibody, while treatment with TNF-α clearly increased the binding between the YTHDF2 and HDAC5 mRNAs (Fig. 7D). Moreover, we found that the knockdown of YTHDF2 significantly reduced the LIF-SE signals of BRD4 and H3K27ac, the expression of LIF, and the degradation rate of HDAC5 mRNA in TNF-α-treated MSCs (Fig. 7E–H). These findings further indicate that YTHDF2 can bind to m6A-modified HDAC5 mRNA and promote its degradation. To further explore the specific m6A sites on HDAC5 mRNA, we predicted m6A modifications via SRAMP software [26]. Two high-confidence m6A sites were predicted to be located in the CDS region of HDAC5 mRNA: site 1 at Chr17: 44082589 and site 2 at Chr17:44092438 (Fig. 7I). SELECT-qPCR was performed to detect the m6A modification level at these sites, and the results indicated that after TNF-α stimulation, the m6A modification level at site 1 increased, whereas it decreased after WTAP was knocked down. However, site 2 did not significantly change after TNF-α stimulation or WTAP knockdown (Fig. 7J).

To further confirm the roles of these two potential m6A modification sites, we employed a catalytically inactive type VI-B Cas13 enzyme fused with ALKBH5 (dCas13b-A5) for specific demethylation of HDAC5 mRNA. We designed gRNA-1 (targeting site 1), gRNA-2 (targeting site 2), and a non-targeting gRNA (NT-gRNA), respectively, each targeting sequences approximately 100 nt upstream of the respective site (Fig. 7I). The demethylation effect of gRNA-1 on site 1 was confirmed using the SELECT-qPCR, whereas gRNA-2 had no impact on the m6A level of site 2 (Fig. 7K), possibly because site 2 either does not exist or exhibits only very low levels of m6A modification. RIP-qPCR showed that dCas13-A5 with gRNA-1 significantly reduced the binding of YTHDF2 to HDAC5 mRNA (Fig. 7L). We observed that dCas13b or dCas13b-A5 did not affect HDAC5 expression. However, dCas13b-A5 with gRNA-1 significantly upregulated HDAC5 expression at both the mRNA and protein levels in MSCs treated with TNF-α, while gRNA-2 had no effect (Fig. 7M, N), suggesting that the increase in HDAC5 expression might be associated with weakened YTHDF2 binding. Similarly, we found that only transfection with dCas13b-A5 and gRNA-1, but not gRNA-2, significantly reversed the TNF-α-induced impairment of immunosuppressive function in MSCs (Fig. 7O). Our results indicated that YTHDF2 is the key m6A reader that recognises specific m6A modification sites on HDAC5 mRNA to promote its degradation.

## Discussion

In this study, we found that TNF-α impairs the immunosuppressive function of MSCs and weakens their therapeutic potential via two epigenetic mechanisms: m6A RNA modification and SEs. Specifically, TNF-α induces the upregulation of the m6A methyltransferase WTAP, which decreases HDAC5 expression by promoting its m6A modification and reducing its mRNA stability. Decreased HDAC5 led to elevated H3K27ac levels and the redistribution of SEs, ultimately driving the increased expression of LIF and diminishing the MSC-mediated suppression of T-cell proliferation. The overexpression of HDAC5 effectively promoted the therapeutic effect of MSCs in an inflammatory model with high TNF-α concentrations in vivo. This intricate mechanism, which bridges the post-transcriptional regulation of m6A with the transcriptional amplification of SEs, provides a novel epigenetic perspective on how inflammatory signals can compromise the therapeutic efficacy of MSCs.

As pluripotent stem cells with immunoregulatory functions, MSCs have been widely used for the treatment of inflammatory diseases, and a diverse array of clinical trials have been conducted [27]. However, the clinical efficacy of MSC treatment is not as favourable as expected in some diseases and is also disparate in different patients with a common disease [28, 29]. Recently, several studies have indicated that the limited efficacy of MSCs may result from the complex and discrepant inflammatory microenvironment in these patients [30] because the immunoregulatory functions of MSCs are regulated by various kinds of cytokines [31]. In our study, we found that TNF-α treatment at a relatively high concentration of 400 ng/ml, rather than IL-17a or LPS treatment, significantly impaired the immunoregulatory effects of MSCs on T cell proliferation. TNF-α is one of the most critical inflammatory cytokines in vivo and predominantly promotes the development of inflammatory diseases [32]. In addition, TNF-α plays a critical role in regulating the functions and status of MSCs, specifically impeding the immunosuppressive function of MSCs [5, 33], and the diverse effects of TNF-α on MSCs are dependent mainly on their concentration in vitro and in vivo [34]. Although several studies have shown that TNF-α can promote the immunoregulatory functions of MSCs [35], we suggest that this discrepancy may be due to the different concentrations of TNF-α used in these studies, as the concentration we used represented a greater level of inflammation in vivo.

LIF is the most pleiotropic member of the IL-6 cytokine superfamily. Previous studies have indicated that LIF plays a complex and broad role in regulating T-cell proliferation and differentiation, promoting Treg proliferation but inhibiting Th17 differentiation [36, 37]. Our findings are consistent with a previous report that LIF expression is under the control of an SE in microglia, further supporting the existence of a potent, conserved regulatory module near the LIF gene locus [38]. In addition, MSCs were shown to inhibit the mixed lymphocyte reaction by secreting LIF [39]. However, our results revealed that LIF expression in MSCs increased after TNF-α treatment and that knocking down LIF or adding a LIF-neutralising antibody restored the ability of TNF-α to impair MSC immunosuppression. We suggest that this discrepancy in the effect of LIF on T-cell proliferation is due to the different experimental systems used in the studies, indicating the pleiotropic effect of LIF in the inflammatory environment. Specifically, LIF is associated with the severity of inflammatory diseases, including ankylosing spondylitis (AS) [40] and RA [41], which are characterized by high TNF-α levels and are positively correlated with inflammatory markers, such as C-reactive protein (CRP) and the erythrocyte sedimentation rate (ESR) [42, 43], indicating a proinflammatory effect of LIF in these diseases.

SEs, as critical epigenetic regulatory mechanisms, consist of clusters of TEs with a high density of TFs and cofactors, facilitating the transcription of target genes [8]. In the inflammatory microenvironment, the formation and distribution of SEs, as determined by their markers, including H3K27ac, are strongly altered, thereby affecting various functions of cells [44, 45]. Previous studies reported that the differentiation potential of MSCs is under the precise control of SEs, but whether SEs contribute to the regulation of immunoregulatory function is largely unclear [10]. For the first time, we demonstrated that SE regulates LIF expression and the subsequent immunoregulatory function of MSCs and that the formation and distribution of this SE are affected by the inflammatory microenvironment, especially the critical cytokine TNF-α. In addition, this effect was caused by TNF-α-mediated downregulation of an HDAC for H3K27ac, HDAC5. Similarly, HDAC5, which is suppressed by TNF-α, was identified as a novel inflammatory mediator in RA [46], suggesting the critical role of HDAC5 in either promoting inflammation development or impeding the therapeutic effect of MSCs in inflammatory diseases. In this study, we confirmed that MSCs overexpressing HDAC5 had a better therapeutic effect than unmodified MSCs in an inflammatory arthritis model with high levels of TNF-α in vivo. Strategies rescuing HDAC5 expression may be a way to circumvent the negative effects of the inflammatory microenvironment on the therapeutic effect of MSCs and have therapeutic applications in inflammatory disease treatment.

m6A modification is another critical epigenetic mechanism that regulates gene expression through translation, RNA splicing, and mRNA degradation and is recognised as a key regulator of cell function and disease progression [47]. WTAP, a m6A writer, was reported to be elevated in response to a variety of inflammatory stimuli, such as TNF-α, in a manner dependent on NF-κB p62, and in many inflammatory diseases [23, 48]. Similarly, we observed TNF-α-mediated upregulation of WTAP expression in MSCs. We propose that this likely occurs through a mechanistically conserved regulatory pathway. Furthermore, our study demonstrated that WTAP-mediated m6A modification represents a major mechanism by which TNF-α treatment decreased HDAC5 expression, which reduced mRNA stability and promoted mRNA degradation. Several studies have shown that SEs act as regulators of m6A modification. For example, the SE-driven m6A readers IGF2BP2/3 stabilise DDX21 mRNA to facilitate the progression of acute myeloid leukaemia [49], and the SE-regulated lncRNA LINC01089 induces alternative splicing of DIAPH3 through m6A modification to drive hepatocellular carcinoma progression [50]. We detected no substantial alterations in the expression levels of m6A-related enzymes after HDAC5 interference or overexpression, indicating that the regulatory relationship between SEs and m6A modification is not constant. Since SEs and m6A independently contribute to the modulation of gene expression at both the transcriptional and posttranscriptional levels, we suggest that there is an interdependent regulatory relationship between them and that these regulatory networks may differ in cells or even in the same cell under different environments.

As a highly pleiotropic inflammatory cytokine, TNF-α affects MSC function through multiple pathways beyond a single mechanism. For instance, TNF-α has been shown to promote senescence and apoptosis in MSCs via activation of NF-κB and MAPK signalling [51]. Other studies report that TNF-α regulates MSC quiescence and activation through the p65–CYP7B1–Notch3 axis [52], and enhances MSC migration via m6A-dependent regulation of ELMO1 [17]. In line with these multifaceted roles, our study demonstrates that perturbation of the WTAP–m6A–HDAC5 axis significantly—yet not fully—reverses the effects of TNF-α. This partial phenotypic rescue implies that although WTAP-mediated m6A methylation in HDAC5 plays a central role in TNF-α-driven functional alterations, additional signalling mechanisms are likely involved. The identity and magnitude of contribution of these parallel pathways remain to be elucidated. Thus, the m6A signalling pathway uncovered here represents one important, but not exclusive, mechanism through which TNF-α modulates MSC biology under inflammatory conditions.

## Conclusion

This study revealed that TNF-α impairs the immunoregulatory capacity of MSCs to inhibit T-cell proliferation via RNA m6A modification and SEs, which may provide new evidence for optimising therapeutic outcomes in inflammatory diseases. There are several limitations in our present study. Our study focused on the WTAP-m6A-HDAC5 axis; however, TNF-α triggers multiple inflammatory pathways that may independently or synergistically regulate MSC immunomodulation. How did these pathways contribute to TNF-α effects? The modified MSC infusion significantly increased the therapeutic effects in SKG mice, but can the same modifications yield similar results in diseases with different inflammatory microenvironments? Alternatively, could distinct microenvironments benefit from the use of differently modified MSCs to achieve optimal therapeutic outcomes? Furthermore, regarding human treatments, will variations in microenvironments affect treatment efficacy? These intriguing questions need further investigation in future research.

## Supplementary information

Supplemental figures and tables

## Data Availability

The datasets generated in the present study are deposited in the NCBI Gene Expression Omnibus (GEO) and are available in the GSE284785 (secure token: ahkbkkaqxfkpvsx) and GSE284786 datasets (secure token: gjsjmqkkdzwtrgf). The code used in this study are available from the corresponding author upon reasonable request.
