# Supplementary material for: TNF-α-driven m6A modification disrupts the immunoregulatory function of MSCs by regulating HDAC5-dependent super-enhancers
Source: Cell Death Dis. 2025 Dec 23;16(1):902. doi: 10.1038/s41419-025-08192-w (PMC12728190; doi:10.1038/s41419-025-08192-w)
Supplement: Supplementary file 1 — Supplemental figures and tables [file 41419_2025_8192_MOESM1_ESM.pdf]

Supplemental materials for

TNF-α-Driven m6A Modification Disrupts the Immunoregulatory Function of MSCs by Regulating HDAC5-Dependent Super-Enhancers

Weihao Zhang[1#], Jiajie Lin[1#], Yi Zhou[1#], Changhua Wu[1], Qibo Li[1], Junhua Chen[1], Yipeng Zeng[1], Zipeng Xiao[1], Huiyong Shen[1], Yanfeng Wu[2], Zepeng Su[1*], Wenhui Yu[1*], Zhongyu Xie[1, 2*]

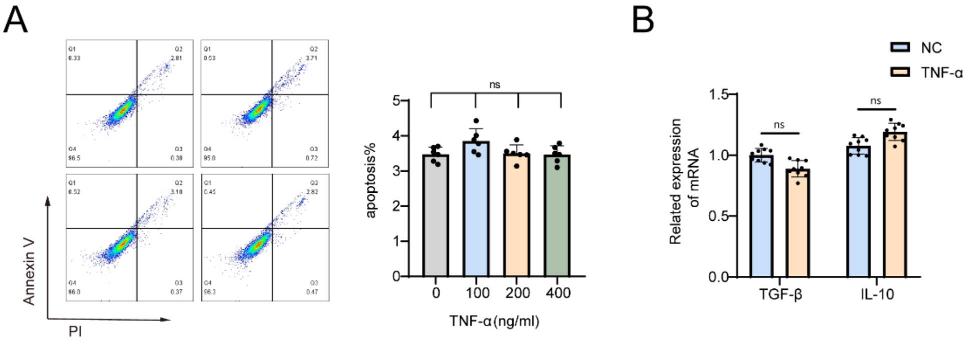

Supplementary Fig. 1: Effects of TNF-α on apoptosis and expression of IL-10/TGF-β in MSCs

(A) Flow cytometry analysis of apoptotic cells in MSCs treated with different concentrate of TNF-α.

(B) qPCR analysis showing the mRNA expression levels of TGF-β and IL-10 in MSCs treated with or without TNF-α.

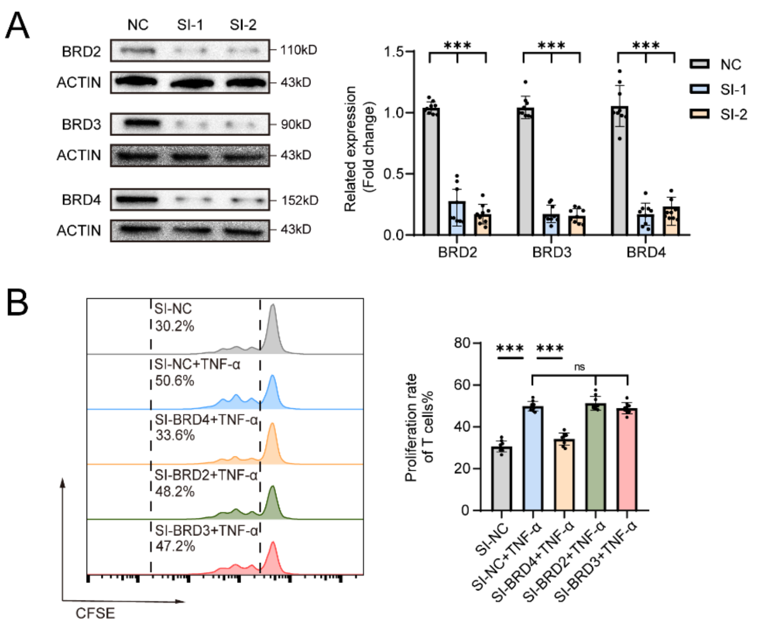

Supplementary Fig. 2: Knockdown of BRD4 restores the immunosuppressive capacity of MSCs impaired by TNF-α

(A) Western blot analysis showing the BRD2, BRD3 and BRD4 expression levels in MSCs treated with SI-NC, SI-1 or SI-2 for BRD2, BRD3 or BRD4.

(B) Proliferation rates of T cells cocultured with MSCs treated with SI-NC, SI-NC+ TNF-α, SI-BRD4+ TNF-α, SI-BRD2+ TNF-α, or SI-BRD3+ TNF-α.

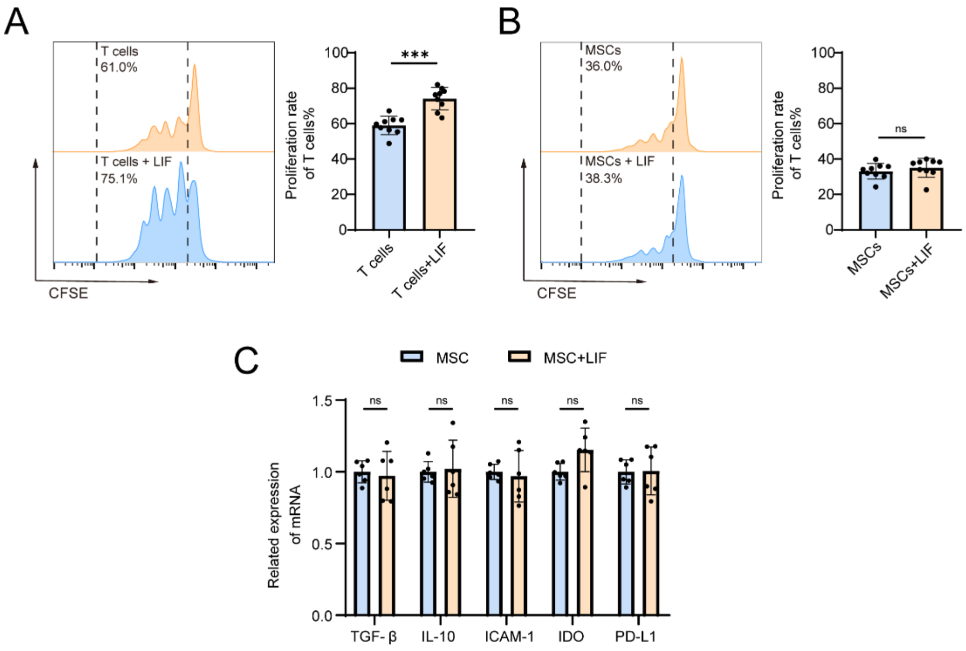

Supplementary Fig. 3: LIF directly promotes T cell proliferation

(A) Proliferation rates of T cells alone or treated with recombinant LIF (10ng/ml).

(B) Proliferation rates of T cells cocultured with MSCs alone or pretreated with recombinant LIF for 48 hours.

(C) qPCR analysis showing the mRNA expression levels of TGF-β, IL-10, PGE2, IDO and PD-L1 in MSCs pretreated with recombinant LIF.

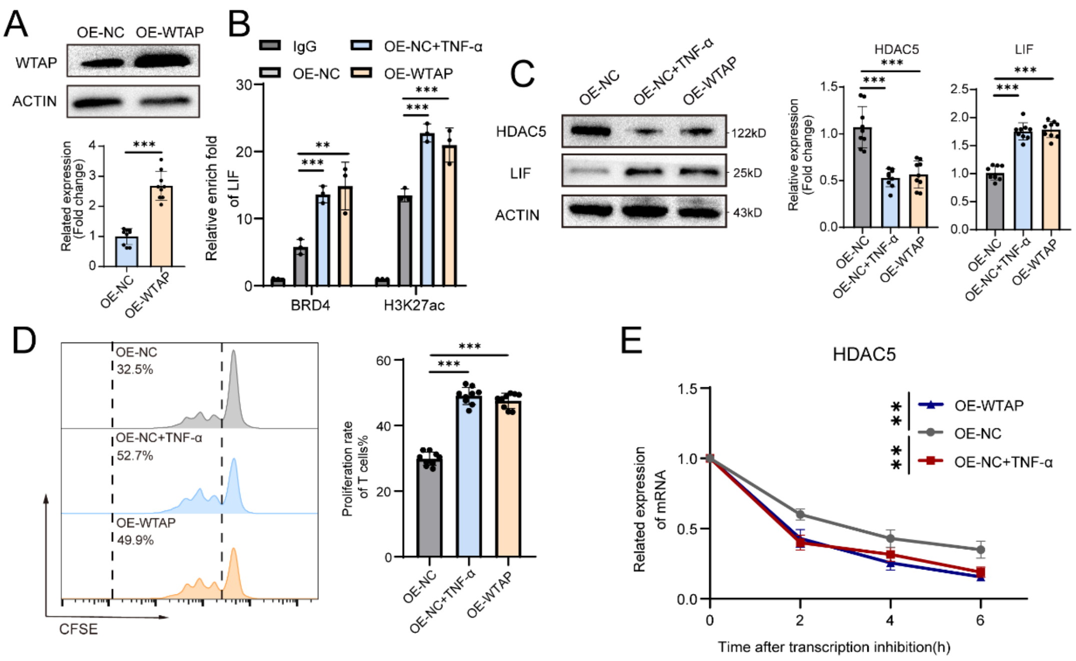

Supplementary Fig. 4: Overexpression of WTAP mimics a similar phenotype in MSCs treated with TNF-α

(A) Western blot analysis showing the WTAP expression levels in MSCs treated with OE-NC or OE-WTAP.

(B) CUT&Tag-qPCR showing signals of BRD4 and H3K27ac at the LIF locus in MSCs treated with OE-NC, OE-NC+TNF-α or OE-WTAP.

(C) Western blot analysis showing the HDAC5 and LIF expression levels in MSCs treated with OE-NC, OE-NC+TNF-α or OE-WTAP.

(D) Proliferation rates of T cells cocultured with MSCs treated with OE-NC, OE-NC+TNF-α or OE-WTAP.

(E) Degradation rates of HDAC5 mRNA in MSCs treated with OE-NC, OE-NC+TNF-α or OE-WTAP.

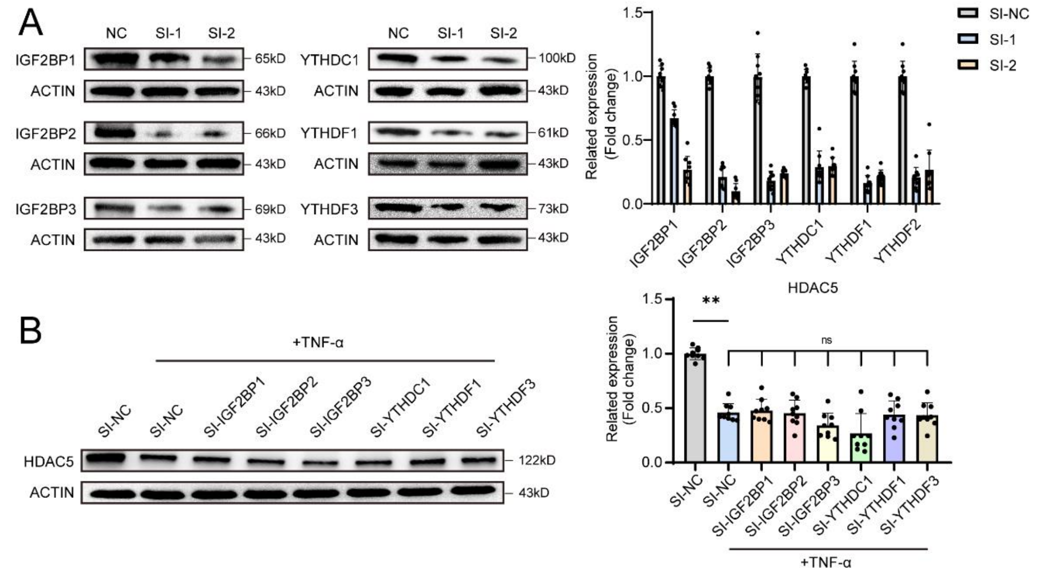

Supplementary Fig. 5: Knockdown of IGF2BP1/2/3, YTHDF1/3, and YTHDC1 does not restore HDAC5 expression

(A) Western blot analysis showing the expression levels of IGF2BP1, IGF2BP2, IGF2BP3, YTHDC1, YTHDF1 and YTHDF3 in MSCs treated with SI-NC, SI-1 or SI-2 for IGF2BP1, IGF2BP2, IGF2BP3, YTHDC1, YTHDF1 or YTHDF3.

(B) Western blot analysis showing the HDAC5 expression levels in MSCs treated with SI-NC and TNF-α-primed MSCs treated with SI-IGF2BP1, SI-IGF2BP2, SI-IGF2BP3, SI-YTHDC1, SI-YTHDF1 or SI-YTHDF3.

Supplemental Table S1: Sequence of siRNAs

	Sense (5'-3')
SI-LIF-1	CCAUACGCCACCCAUGUCATT
SI-LIF-2	CCAACAACCUGGACAAGCUTT
SI-HDAC5-1	GGACUUCUCUGCACAGCAUTT
SI-HDAC5-2	ACACGUUCAUGCUAAAGCATT
SI-WTAP-1	GGCAAGAGATGAGTTAATTTT
SI-WTAP-2	AAGAGTGTACTACTCAAATTT
SI-YTHDF2-1	GGUGAAGCUGCUUGGUCUATT
SI-YTHDF2-2	GCACAGAAGUUGCAAGCAATT
SI-YTHDC2-1	GCGACUCAACAAUGGCAUATT
SI-YTHDC2-2	GGAUUUGAUCAUGCAUCUUTT
SI-YTHDF1-1	GCACTGACTGGTGTAACCTTT
SI-YTHDF1-2	CCCGAGAGUUUGAGUGGAATT
SI-YTHDF3-1	GGUGGAUUUCACCAGUUAATT
SI-YTHDF3-2	AGAUGGUGUAUUUAGUCAATT
SI-YTHDC1-1	CCAGAAGATTATGATATTATT
SI-YTHDC1-2	CCGCGTGAGAATTGGCTTATT
SI-IGF2BP1-1	CCCAGUAUGUGGGUGCCAUTT
SI-IGF2BP1-2	CCAAAGUUCGUAUGGUUAUTT
SI-IGF2BP2-1	CUCUCGGGUAAAGUGGAAUTT
SI-IGF2BP2-2	GUCAACGUCACAUAUGCAATT
SI-IGF2BP3-1	GCUGCUGAGAAGUCGAUUATT
SI-IGF2BP3-2	CGGUGAAUGAACUUCAGAATT

Supplemental Table S2: Sequence of Primers for qPCR

Gene	Forward primer (5'-3')	Reverse primer (5'-3')
GAPDH	GGAGCGAGATCCCTCCAAAAT	GGCTGTTGTCATACTTCTCATGG
LIF	TCTTGGCGGCAGTACACAG	CGACTATGCGGTACAGCTCC
HDAC9	ATGGTTTCACAGCAACGCATT	ACCTTGCCTAAGCGTCTGC
MAP3K7CL	GTTGGCTACTGGCGATTGGAT	TCTTCTGCTATTTGGCAGTGC
BRCA2	TGCCTGAAAACCAGATGACTATC	AGGCCAGCAAACTTCCGTTTA
HDAC5	TGAACCCAACTTGAAAGTGCG	CGCTGTTACACACGGACGA
WTAP	CTTCCCAAGAAGGTTCGATTGA	TCAGACTCTCTTAGGCCAGTTAC
METTL3	ATTTTCCGGTTAGCCTTCGGG	TGGATTCCGTAGATCCAAGTGC
METTL14	GAACACAGAGCTTAAATCCCCA	TGTCAGCTAAACCTACATCCCTG
FTO	GAATTCTATCAGCAGTGGCAGC	GGATGCGAGATACCGGAGTG
ALKBH5	CGGCGAAGGCTACACTTACG	CCACCAGCTTTTGGATCACCA
YTHDF2	GAGACCAAAAGGTCAAGGAAACA	GCAGTATATGCATTATTGGGCCTTG
YTHDC2	CGATACGGGGACCAGAGAGA	GGTCATCATTGCATGAGCTGTT
TGF-β	GGCCAGATCCTGTCCAAGC	GTGGGTTTCCACCATTAGCAC
IL-10	GACTTTAAGGGTTACCTGGGTTG	TCACATGCGCCTTGATGTCTG
ICAM-1	ATGCCCAGACATCTGTGTCC	GGGGTCTCTATGCCCAACAA
IDO	GCCAGCTTCGAGAAAGAGTTG	ATCCCAGAACTAGACGTGCAA
PD-L1	TGGCATTTGCTGAACGCATTT	TGCAGCCAGGTCTAATTGTTTT
SELECT HDAC5-site1	CTGAGCGGAGAAGCAGTCC	GCTGTTACACACGGACGACG
SELECT HDAC5-site2	ACCGCAAAACTCCTACAGCA	TGTAGAGCACAGAGGGGTCA
